# Algal sorbents and prospects for their application in the sustainable recovery of rare earth elements from E-waste

**DOI:** 10.1007/s11356-023-27767-8

**Published:** 2023-05-25

**Authors:** João Pinto, João Colónia, Azadeh Abdolvaseei, Carlos Vale, Bruno Henriques, Eduarda Pereira

**Affiliations:** 1 Department of Chemistry, University of Aveiro, Aveiro, Portugal; 2 LAQV-REQUIMTE – Associated Laboratory for Green Chemistry, University of Aveiro, Aveiro, Portugal; 3 CIIMAR – Interdisciplinary Centre of Marine and Environmental Research, Matosinhos, Portugal

**Keywords:** Biosorption, Recycling, Critical raw materials, Algae, Sorption, Carbon neutrality, Biorefinery

## Abstract

Efficient and sustainable secondary sourcing of Rare-Earth Elements (REE) is essential to counter supply bottlenecks and the impacts associated with primary mining. Recycled electronic waste (E-waste) is considered a promising REE source and hydrometallurgical methods followed by chemical separation techniques (usually solvent extraction) have been successfully applied to these wastes with high REE yields. However, the generation of acidic and organic waste streams is considered unsustainable and has led to the search for “greener” approaches. Sorption-based technologies using biomass such as bacteria, fungi and algae have been developed to sustainably recover REE from e-waste. Algae sorbents in particular have experienced growing research interest in recent years. Despite its high potential, sorption efficiency is strongly influenced by sorbent-specific parameters such as biomass type and state (fresh/dried, pre-treatment, functionalization) as well as solution parameters such as pH, REE concentration, and matrix complexity (ionic strength and competing ions). This review highlights differences in experimental conditions among published algal-based REE sorption studies and their impact on sorption efficiency. Since research into algal sorbents for REE recovery from real wastes is still in its infancy, aspects such as the economic viability of a realistic application are still unexplored. However, it has been proposed to integrate REE recovery into an algal biorefinery concept to increase the economics of the process (by providing a range of additional products), but also in the prospect of achieving carbon neutrality (as large-scale algae cultivation can act as a CO_2_ sink).

**Graphical abstract:**

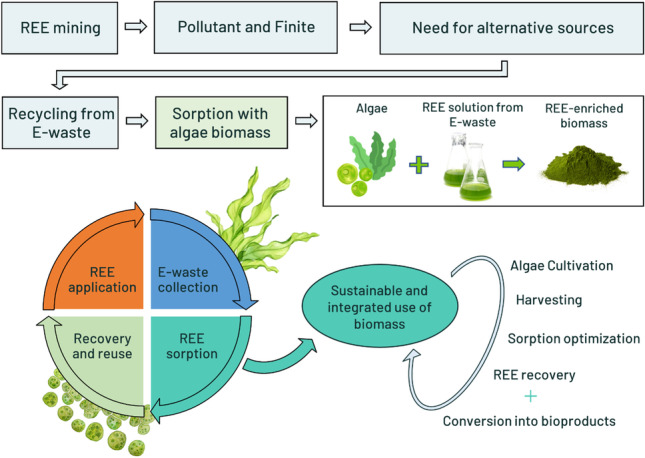

## Introduction

The sustainable management of the planet’s natural resources requires a holistic approach in which sustainable consumption and production pave the way for economic, social, and technological progress, supporting the needs of present and future generations. Since global environmental changes and population growth are pushing the need for sustainable development, urgent measures must be taken to prevent the depletion of available resources (Kobayashi and Nakajima 2021). To this end, the United Nations published the *2030 Agenda for Sustainable Development* that identifies modern societal, economical, and environmental challenges for sustainable development. Following this agenda, industries have been encouraged to pursue innovative technologies which can contribute to sustainable development through the mitigation of carbon emissions and the use of efficient and renewable energies (Fuso Nerini et al. 2019). However, the production of modern “green” technologies such as electric vehicles and wind turbines is dependent on the use of valuable and scarce materials. These materials have been classified as “Critical Raw Materials” (CRM) by the European Commission. While they are essential to the production of clean energy, their value is also closely tied to their applications in high-tech and emerging technologies. Examples include the use of Lithium in the batteries of electric vehicles, the use of Platinum Group Elements (PGE) in catalytic converters and the use of Rare Earth Elements (REE) in fibre optics, magnets, and electronic devices (smartphones, computers, etc.). Such technological advances have led to increasing demand for CRM on a global scale. Their use is expected to double between 2010 and 2030 while their supply remains constrained by geopolitical factors (Mathieux, F. et al. 2018). Such an imbalance between supply and demand can lead to price shifts and threaten the development of modern technologies. The economic importance and supply risk of these materials are thus the determining factors which contribute to their criticality (Hofmann et al. 2018). Economic importance is built on the allocation of raw materials in industrial applications while supply risk gauges the global production, sourcing, importation reliance and governance of supplier countries (European Commission 2020). The supply of many of these materials is concentrated in a small number of countries. For example, the vast majority (70 – 90%) of most PGE is supplied by South Africa. Another severe scenario refers to the REE, for which China is responsible for 58% of primary mining on a global scale. Furthermore, China is also responsible for processing REE from other countries, dominating the global supply of processed REE at 80% (Pawar and Ewing 2022). The supply bottlenecks of these critical materials are therefore a major challenge for achieving UN Sustainable Development Goal 12: ensure sustainable consumption and production patterns. However, there is increasing interest in researching alternative sources of these CRM (Giannetti et al. 2020).

Studies have shown how recycling (Santillán-Saldivar et al. 2021) and working towards a circular economy (Dantas et al. 2021) can contribute to better managing critical materials such as the REE. The present paper aims to review the existing methods for recovering REE from secondary sources (mainly from electronic waste, E-waste), as well as to examine the potential of algal-based sorbents to remove/preconcentrate these elements from aqueous solutions. Several sorbent and solution-intrinsic aspects were considered, and the advantages and disadvantages of conventional and algal-based sorbents were explored.

### Alternative sources of critical raw materials: the case of electronic waste

In principle, sourcing of Critical Raw Materials such as REE is possible through the mining and processing of natural ores or through the recovery of these metals from end-of-life products such as E-waste (Parajuly et al. 2020). The total value of the raw materials contained in E-waste is estimated to exceed 55 billion euros (Baldé et al. 2017). Because of this, the role of the E-waste recycling industry in terms of circular economy is becoming increasingly important. Closing the loop of such products while recovering valuable materials during their processing could promote economic growth and reduce the over-exploration of the available natural resources, thus contributing to sustainable development in line with United Nations’ expectations (Bressanelli et al. 2020). To improve this, several organizations have proposed initiatives and legislation to better manage end-of-life electronic equipment. For example, the European WEEE directive, the Australian National Waste Policy and the Japanese Home Appliance Recycling Law improved the collection rates and efficiency of the recycling process, while others such as the RoHS directive placed restrictions on the use of certain substances in electronic equipment (Kumar et al. 2017). Despite this, the ever-growing waste stream of electronic equipment has not been accompanied by a sufficient increase in recycling rates. As previously compiled by Kumar et al. (2017), unsatisfactory E-waste recycling practices have been reported worldwide in Europe (Van Eygen et al. 2016), Africa (Abdelbasir et al. 2018), America (Li et al. 2015), Oceania (Gough 2016) and Asia (Yoshida et al. 2016). Despite a 38% increase in E-waste between 2010 and 2019, less than 20% (8.9 million metric tonnes) were properly recycled (Baldé et al. 2017; Forti et al. 2020; United Nations 2020). It is estimated that only 20% of annual E-waste is formally recycled, while most of the remaining waste is dumped in landfills (Ilankoon et al. 2018). High recycling costs lead countries to export their waste to developing countries, where dismantling occurs under unregulated and dangerous conditions (Tansel 2017). Since these wastes can contain significant concentrations of persistent contaminants such as Mercury (Hg), Cadmium (Cd) and Lead (Pb), these landfills can act as sources of contamination for soil and groundwater (Rodríguez-Padrón et al. 2020). Open-air landfills are the most common, where incineration and runoff can lead to contamination of nearby water streams (Man et al. 2013; Cesaro et al. 2019). Given the serious disadvantages of improper handling, formal recycling is the safest way to manage E-waste and therefore needs to be encouraged (Ahirwar and Tripathi 2021).

### Transformation and recovery of critical raw materials from E-waste

Besides environmental issues, severe economic losses can also result from inadequate optimization of E-waste recycling techniques. Apart from the high precious metals content in E-waste, their purity can be up to ten times higher than that of extractable ores (Islam et al. 2020). Scientific publications have extensively demonstrated the benefit of capitalizing on the recovery of precious metals from E-waste and lessening the environmental impacts associated with E-waste processing. However, there is still a lack of environmentally friendly and efficient techniques for their recovery (Rodríguez-Padrón et al. 2020).

The recycling of E-waste can be summed up in three distinct phases: disassembly, mechanical separation and extraction of precious metals and their subsequent refinement (Cui and Zhang 2008). Disassembly is essential to separate metal-bearing or hazardous parts of the waste. Mechanical separation involves crushing and magnetic separation of the metallic and non-metallic fractions. This particular step is generally cost-effective and environmentally friendly (Huang et al. 2009; Lu and Xu 2016). However, further extraction of precious metals (mainly focused on the recovery of gold and noble metals) requires specific, expensive, and often less sustainable methods. In general, pyrometallurgical and hydrometallurgical methods are primarily considered. In pyrometallurgical (or thermal) processing, E-waste undergoes smelting, melting, sintering or gas phase reactions at high temperatures (Huang et al. 2009). Pyrometallurgical recycling facilitates scalability and uses few chemicals (Assefi et al. 2020) but releases harmful and toxic gases such as dioxins and carbon dioxide, and volatile metals like Hg (Lu and Xu 2016). Currently, hydrometallurgical processes are preferred over pyrometallurgy for the recovery of precious metals from E-waste (Akcil et al. 2015; Rodríguez-Padrón et al. 2020). Aside from low emissions, hydrometallurgical recycling yields high recovery rates, minimal slag generation, consumes less energy and offers easier working conditions (Lu and Xu 2016). The first step in a hydrometallurgical process involves the leaching of the precious metals present in the waste which, although efficient, generally leads to the generation of large amounts of wastewater. Common leaching agents include hydrochloric acid (HCl), nitric acid (HNO_3_) and sulfuric acid (H_2_SO_4_), aqua regia (3 HCl:HNO_3_), hydrogen peroxide, cyanide, iodine, thiourea, halides, thiosulphate, among others (Guo et al. 2015; Islam et al. 2020; Rodríguez-Padrón et al. 2020). After the hydrometallurgical treatment, several purification processes can then be used to recover the precious metals present in the leachates. The main purification methods are based on solvent extraction, adsorption, electrochemical techniques, ion exchange and chemical precipitation. However, these methods can be polluting, and several obstacles still hamper their application on an industrial scale. Therefore, the development of efficient, low-cost refining techniques that do not endanger the environment is of utmost importance (Lu and Xu 2016).

## Rare-earth elements

The term Rare-earth elements (REE) denotes a group of elements, which includes Yttrium (Y), Scandium (Sc) and the lanthanide (Ln) series: Lanthanum (La), Cerium (Ce), Praseodymium (Pr), Neodymium (Nd), Promethium (Pm), Samarium (Sm), Europium (Eu), Gadolinium (Gd), Terbium (Tb), Dysprosium (Dy), Holmium (Ho), Erbium (Er), Thulium (Tm), Ytterbium (Yb) and Lutetium (Lu) (Cotton 2006).

All 17 elements share chemical similarities, which is why they are often referred to as a group (Wang et al. 2020). Despite this, REE are still generally divided into sub-groups based on atomic weight. The light REE (LREE) comprises the elements La-Eu, while the elements Gd-Lu along with Y encompass the heavy REE (HREE). Since Sc does not occur in the same deposits as the remaining elements, it is not included in any of these categories (Dushyantha et al. 2020). Additionally, one of the REE, Pm, is absent from ores because it is very radioactive, and all of its isotopes have relatively short half-lives (Du and Graedel 2013). There is no consensus on the sub-division of the REE, as some authors prefer to include a third sub-group, the medium REE (MREE) with the elements Sm, Eu and Gd (Costis et al. 2021).

The main characteristic that helps to classify these elements as a group is the predominance of their + III oxidation state. Lanthanides follow an electronic configuration in which there is the progressive filling of the 4f shell which is more evident for Ln^3+^ ions (Cotton 2006). In general, atomic radius and atomic mass increase simultaneously due to the attachment of electrons to the outer shells. For the lanthanides, however, the 4f subshells contract along the lanthanide series, and a reduction of atomic and ionic radii are observed. This phenomenon is called the “lanthanide contraction”. The lanthanide contraction is a key factor in lanthanide chemistry as it allows differential chemical reactivity among them, despite a common preconceived notion that these elements are chemically similar (Dushyantha et al. 2020). The stability sequence associated with the contraction is often considered when implementing REE separation and purification methods such as solvent extraction and ionic exchange, as there is an overall increase in complex stability from La^3+^ to Lu^3+^ (Cotton and Raithby 2017).

### Applications and criticality of rare earth elements

Rare earth elements are virtually irreplaceable in modern industry and play a crucial role in fields such as electronics, aerospace engineering and renewable energy (Wang et al. 2015). The chemical specificities of REE grant them great versatility in a vast array of industries. In metallurgy, they are added to steel to increase strength and thermal stability. In the oil industry, they act as catalysts to increase the number of short hydrocarbon molecules in the final product. In ceramics, they can act as coatings and colouring or decolouring glass additives (Preinfalk and Morteani 1989). However, the most notable contribution of REE is generally attributed to their role in a growing number of modern technologies. The use of Nd and Dy in high-performance permanent magnets is a clear example of the irreplaceable nature of REE in these technologies. Rare earth magnets have exceptional magnetic properties coupled with high productivity and affordability and are used in numerous applications such as hard drives, mobile phones, wind turbines and hybrid vehicles (Omodara et al. 2019). Rare earth elements also play a major role in lighting applications, as REE phosphors combine high luminous intensity and precise colour determination with affordability, efficiency and durability (Wang et al. 2011). Other applications of REE also include the aerospace industry, healthcare, battery alloys, catalysts, and polishing powders, among others (Binnemans et al. 2013; Cardoso et al. 2019; Dutta et al. 2016; Haque et al. 2014). Given their wide range of applications, a potential shortage of these materials will permanently jeopardize the Sustainable Development Goals set by the United Nations, as long as the development of modern technologies pushes an increasing demand for REE.

The search for recovery technologies of REE from waste is particularly urgent as their supply risk is higher than any other Critical Raw Material (Keilhacker and Minner 2017). This high level of criticality is not necessarily due to the scarcity of natural reserves. Rare earth elements are usually found in the minerals monazite, xenotime and bastnaesite (Kumar et al. 2010), and mineral resources are considered mineable if their concentration is in the range of 10 to 300 ppm (Achary et al. 2017). Contrary to what the name might suggest, there are an estimated 110 million tons of REE reserves worldwide. Half of these reserves are located in China, the rest are mainly distributed between Russia (17.3%) and the United States (11.8%) (Wübbeke 2013). Smaller deposits can also be found in Brazil, South Africa, Tanzania and Sweden (Soukeur et al. 2021). Despite this, REE production and supply are severely constrained with China being responsible for 80% of all global production and marketing (Massari and Ruberti 2013; Pawar and Ewing 2022). With the evolution of technology in the second half of the twentieth century, world REE production has tripled since 1985 (Wübbeke 2013). The consequences of this supply bottleneck were made clear in 2006, when China imposed exportation restrictions on REE, drastically reducing global supply. The irreplaceability in technologies and the lack of an alternative supplier led to an explosion in REE prices (Keilhacker and Minner 2017). In 2010, import-dependent countries again faced a shortage of REE supplies when China suddenly cut 70% of its export quotas. Since demand was higher than ever before, prices rose by up to 850%, during the so-called Rare-earth crisis (Mancheri 2015).

## Technologies for the recovery of Rare-earth elements from secondary sources

From an availability and sustainability perspective, developing efficient and environmentally friendly alternatives to recover REE from secondary sources is the best way to avoid the continued depletion of these resources. This urgency has been acknowledged by the scientific community and publications on REE recycling have proliferated in the past two decades (Jyothi et al. 2020). Most studies show that the purification and recovery of REE from secondary sources such as E-waste usually occurs after chemical leaching by hydrometallurgical processes, which are not required in the case of in industrial wastewater or acid mine drainage (AMD). For REE, the most common separation technique is solvent extraction, which involves two liquid phases, although techniques involving one liquid and one solid phase, such as ion exchange, membrane separation and sorption have also been used (Chen et al. 2018; Jyothi et al. 2020). Each of these techniques presents a unique set of advantages and disadvantages, which dictate their application in a given scenario. Some of these properties are summarized in Table 1.

**Table 1:** The most common REE recovery technologies and some of their key aspects

Technique	Pros	Cons	References
Solvent extraction	• Technologically simple • Efficient • Fast • Cheap reagents • Can be selective	• Large amounts of consumed reagents • Generation of large amounts of waste • Largen environmental impacts	• Swain and Mishra (2019)
Ion exchange	• High purity yield • Wide availability of ion exchangers • Technologically simple	• Low selectivity and kinetics	• Pereao et al. (2018)
Membrane separation	• Applicable in complex solutions • Low energy requirements • No use of chelating agents	• Low selectivity • Costly	• Ramprasad et al. (2022)
Sorption	• Abundance of sorbents • Fast • Efficient • Technologically simple • Environmentally benign	• High cost of some synthetic materials • Low selectivity • Complex extraction	• Jyothi et al. (2020)
Biosorption	• Cheap • Fast • Abundance of biosorbents • Technologically simple • Environmentally benign	• Low selectivity • Loss of efficiency in multiple cycles • Complex extraction • Minimal waste generation	• Jyothi et al. (2020) • Giese (2020)

### Solvent extraction

Solvent extraction is used to separate metal compounds based on their solubility into two immiscible liquids, usually comprising an aqueous and an organic phase. This technique is widely used in hydrometallurgy because its application does not require sophisticated instrumentation and the chemical reagents used are generally cheap (Swain and Mishra 2019). The organic phase usually consists of an extractant, a diluent and a modifier. Given the chemical and physical similarities among the REE, the efficiency of solvent extraction performance is highly dependent on the extraction efficiency and selectivity of the extractant. An ideal extractant would be able to withstand a wide pH range, and be highly selective and stable throughout the process while being inexpensive and environmentally friendly (Gupta 2003). There is a vast array of extractants that can be used in REE recovery from secondary sources and they are generally classified as cation exchangers, anion exchangers, chelating, solvating and synergistic (Xie et al. 2014). Cation exchangers consist mainly of carboxylic, organophosphorus and glycol amide acids. These extractants are highly efficient and work at low pH (3 – 5), which requires the addition of a base to the aqueous solution to maintain the optimal extraction pH (Hidayah and Abidin 2018); Anion exchangers are used in the presence of strong ionic solvents. These extract metals as anionic complexes and usually consist of long chains of alkali amines. However, these amines must be protonated before use (Gupta 2003; Hidayah and Abidin 2018); Chelating extractants possess a ring structure surrounding the extractant molecule, which acts as a ligand for surrounding ions. The binding mechanism is similar to that of cation exchangers, however chelating extractants are generally unfavourable in REE extraction (Hudson 1982; Hidayah and Abidin 2018); Solvating extractants use the solubility of the inorganic metal species in the organic phase to extract metals by solvation (Swain and Mishra 2019); Finally, synergistic extractants, also known as bi-functional solvents, can be a combination of extractant, modifier, and diluent. Numerous synergistic extractants have been used, including a combination of previous examples such as cation and anion exchangers (Xie et al. 2014). They are efficient and regenerable, but also have disadvantages such as poor contact area and the formation of a third phase (Hidayah and Abidin 2017).

### Ion exchange

Ion exchange is used to obtain high-purity REE for electronic applications. The mechanism of ion exchange involves a reversible exchange of ions between a solid (ion exchanger) and a liquid phase. Depending on the charge of the ions exchanged, the ion-exchanger can be classified as a cation- or anion-exchanger (Levchuk et al. 2018). Ion exchangers have a structure with an excess charge (positive or negative) that is compensated by counter ions with the opposite charge. However, these counter ions are free to move and are easily replaced by other ions present in the liquid phase.

Polymer resins are widely used ion exchangers. Resins are available as gels or microporous structures that can be processed into an acidic cation exchanger or a basic anion exchanger (Levchuk et al. 2018). Recently, other synthetic inorganic exchangers have been used to recover REE. Materials such as hydrous oxides and silicate salts of Titanium (Ti), Zirconium (Zr) and Thorium (Th) have attracted attention in REE extraction due to their high selectivity and stability (Borai et al. 2012).

### Membrane separation

Membrane separation processes are known to be selective and affordable while consuming little energy (Hidayah and Abidin 2018). They have several advantages over solvent extraction as they are freed from the use of large amounts of organic solvents and subsequent problems in phase separation. All membrane processes are based on a non-equilibrium mass transfer through a semipermeable barrier (membrane) into the receiving phase, driven by a concentration gradient or other external forces (Chen et al. 2018).

Processes such as nanofiltration, electrodialysis and reverse osmosis are available, however, the active transport of ions across the membrane varies according to the active membrane layer and the aqueous medium. Nanofiltration has been shown to have great potential in REE recovery, as it has good permeability for singly charged ions and rejection of multiply charged ions (López et al. 2019). Polymer membranes such as Teflon and polyamide membranes are examples used in REE recovery (Hidayah and Abidin 2017). Liquid membranes (emulsion and supported liquid membranes) have also been used, but their instability has hampered their use in industrial applications in favour of polymer membranes.

### Sorption, biosorption and bioaccumulation

Adsorption refers to the binding of an ion in the aqueous phase onto the surface of a solid adsorbent, (Czelej et al. 2016; Soliman and Moustafa 2020). Metal adsorption is generally considered a chemical or physical interaction between metal ions and functional groups such as carboxyl, hydroxyl, amino, and phosphoryl groups present on the sorbent surface (Won et al. 2014). These interactions can include physisorption mechanisms such as electrostatic interactions and Van der Walls forces, or bonding through the surface and inner sphere complexation, surface precipitation and co-precipitation and ion exchange (Tan et al. 2015; Gao et al. 2020). The wide range and availability of adsorbents that can be used have fuelled research on this technique (Anastopoulos et al. 2016). Common adsorbents for REE recovery include silica-based materials, natural or modified zeolites, natural or modified clays, nanocomposites, and hybrid materials, among others (Anastopoulos et al. 2016; Iftekhar et al. 2018; Ahmed et al. 2020). Other materials can also be synthesised and optimized to meet the desired application. Examples include magnetic and non-magnetic materials. Regarding the adsorption of REE ions, nanomaterials are usually based on carbon or silica and display high adsorption ability, which is linked to high porosity and surface area and multi-functionality to bind specific REE. Non-magnetic nanomaterials are more commonly used in REE recovery due to their superior yield and selectivity. However, they lack the facilitated magnetic separation process that is possible when using magnetic nanomaterials (Kegl et al. 2020). After the enrichment of the metal ions in the sorbent, desorption processes are necessary for their reuse.

Besides functionalized materials, sorbents of biological origin have also attracted attention in the recovery of REE. Biosorption is in many respects a nearly identical process to adsorption and can be defined as a process in which a sorbate is concentrated on the surface of a biological matrix (Chojnacka 2010). This process is independent of metabolism and takes place on the surface of the cell wall of inactivated biomass. The possible metal binding mechanisms can then vary depending on the type of biomass, but also include those found in conventional adsorption (Vijayaraghavan and Yun 2008). While desorption is necessary for costly sorbents, this is not necessarily the case for biosorption, as most sorbents have low economic value and are usually considered undesirable wastes in their respective industries (Vijayaraghavan and Yun 2008). Typical biosorbents can include bacteria, fungi, algae, agricultural wastes, industrial by-products, and other biomaterials such as biopolymers. Biosorption has been widely and successfully evaluated in wastewater remediation in the past decades. The high sorption capability and low cost of these biosorbents inspired their use in the recovery of precious metals such as gold and platinum-group elements (Won et al. 2014; Pinto et al. 2021), and in recent years several papers showed the application of biosorption for the recovery of REE (Gupta et al. 2019). Despite this, raw biosorbents still have some disadvantages compared to traditional sorbents such as a lower adsorption capacity and a shorter lifetime (Gadd 2009). However, modifications such as immobilization or acid/alkali pre-treatment have been shown to improve selectivity and adsorption by several times the initial capacity, and offer many other benefits concerning industrial process applications (Gupta et al. 2019).

The use of living organisms presents a unique characteristic that may improve sorption capacity in the form of intracellular accumulation (bioaccumulation). Bioaccumulation is a two-step process in which the first step is identical to biosorption, as the sorbate is adsorbed on the cell wall surface. Secondly, the sorbate is transported through the cell wall by active transport systems and accumulates inside the cell (Sethurajan et al. 2018). A wide variety of (micro)organisms can bioaccumulate REE. Bacteria make up the majority of these organisms, however, studies focusing on the accumulation of REE by aquatic plants, micro- and macroalgae have also recently emerged (Dev et al. 2020). Bioaccumulation has the potential to achieve lower residual concentrations of sorbate since during intracellular accumulation, binding sites on the cell wall surface are released and become available for new adsorption. The continuous growth of biomass can also lead to the creation of new binding sites (Chojnacka 2010).

It can be argued that the many factors affecting the adsorption process are a disadvantage compared to other separation methods. However, under optimal conditions, the yield efficiency of adsorption can exceed that of traditional solvent extraction and ion exchange. Apart from sorbent-intrinsic properties, the main factors that can influence the adsorption process are the pH, ionic strength, temperature, initial metal concentration, sorbent dosage and contact time (Allahkarami and Rezai 2019). The pH of the solution not only influences the metal speciation but can also affect the surface charge of the sorbent (Vijayaraghavan and Yun 2008; Awual et al. 2015). In addition, when the ionic strength is increased, the elements can compete for the binding sites of the sorbent. Regarding the characteristics of the aqueous phase, the initial metal concentration is also of great importance, as it dictates the concentration gradient, a driving force of mass transport (Awual et al. 2013; Allahkarami and Rezai 2019). Sorbent dosage and contact time can affect adsorption efficiency as they affect the frequency and availability of binding sites. Typically, a sharp increase in removal driven by the strong concentration gradient is observed in the initial contact times, followed by a gradual decrease and eventual plateau as the contact time is extended (Allahkarami and Rezai 2019). For economic and environmental reasons, it is ideal to reduce the sorbent dosage without compromising the results, as this reduces costs and reduces the amount of waste generated at the end of the adsorption process.

### Recovery of Rare-earth elements through algae

With the search for environmentally sound methods of waste recycling and the concern to reduce anthropogenic impacts on the environment, biological technologies have shown to be a promising alternative to conventional recycling methods. Natural resources are now considered to be ecologically safer, cheaper and more efficient alternative materials in areas such as the remediation of contaminated waters (Bilal et al. 2018). These benefits have also inspired their use in recycling technologies and the recovery of precious metals from secondary sources. Among the several biological materials that can be applied in recovery technologies, algae have received increasing scientific focus.

With regards to REE recovery, parameters such as pH and ionic strength influence the sorption process (as in normal adsorption processes), but the algal conditions are also a major factor that distinguishes these studies from the typical conventional sorbents. In the existing literature, a wide variety of algae species, pre-treatments and biomass states (dried and live) have been used to remove persistent pollutants such as heavy metals from contaminated waters. However, the influence of these parameters on the recovery of REE is still poorly understood. Table 2 presents a compilation of existing studies using algal sorbents to recover REE from aqueous solutions. Parameters relating to the sorbent used (organism), the properties of the aqueous phase and the maximum sorption capacity were considered.

**Table 2:** Experimental parameters of published studies involving sorption of Rare-Earth Elements by algae

Reference	Organism					Aqueous medium					Sorbent dosage	Maximum sorption capacity
	Species	Size	Treatment	Living	Dried	Elements	Concentration range	Mono	Multi	pH		
Birungi and Chirwa 2014	*D. multivariabilis, S. acuminutus, C. saccharophilum, S. bacillaris*	Micro	–	–	X	La	15–150 mg L^−1^	X	–	6	0.5 g L^−1^	100 mg g^−1^
Costa et al. 2020a	*U. lactuca, U. intestinalis, F. spiralis, F. vesiculosus, Gracilaria sp., O. Pinnatifida*	Macro	–	X	–	Y, La, Ce, Pr, Nd, Eu, Gd, Dy, Tb	89–163 μg L^−1^	–	X	8	3 g L^−1^	48.5 μg g^−1^
Costa et al. 2020b												
Diniz and Volesky 2005	*S. polycystum*	Macro	Ca loading in a solution of CaNO_3_ for 24 h	–	X	La, Eu, Yb	61–1245 mg L^−1^	X	X	3–5	2 g L^−1^	La: 139 mg g^−1^ Eu: 152 mg g^−1^ Yb: 156 mg g^−1^
Fabre et al. 2021	*Ulva* sp., *Gracilaria* sp.	Macro	–	X	–	Nd	10–500 μg L^−1^	X	–	8.2	0.5–5.5 g L^−1^	1.87 mg g^−1^
Ferreira et al. 2020	*U. lactuca, F. spiralis, Gracilaria sp.*	Macro	–	X	–	Gd	10, 150, 500 μg L^−1^	X	X	8	3 g L^−1^	696 µg g^−1^
Ferreira et al. 2021	*Ulva* sp., *Gracilaria* sp.	Macro	–	X	–	Dy	10–500 μg L^−1^	X	–	8.0	0.5–5.5 g L^−1^	1.70 mg g^−1^
Henriques et al. 2021	*U. lactuca, U. intestinalis, F. spiralis, F. vesiculosus, Gracilaria sp., O. Pinnatifida*	Macro	–	X	–	Eu	10–500 µg L^−1^	X	–	8.0	3 g L^−1^	827 µg g^−1^
Iovinella et al. 2022	*G. sulphuraria*	Micro	–	X	–	Y, Ce, Eu, Tb	16–28 mg L^−1^	X	X	2.5–5.5	N/A	Y: 2.96 mg g^−1^ Ce: 4.61 mg g^−1^ Eu: 6.54 mg g^−1^ Tb: 5.74 mg g^−1^
Jacinto et al. 2018	*G. gracilis*	Macro	–	X	–	Y, Ce, Nd, Eu, La	500 µg L^−1^	X	X	7.8	2.5 g L^−1^	Y: 740 μg g^−1^ La: 704 μg g^−1^ Ce: 631 μg g^−1^ Nd: 820 μg g^−1^ Eu:729 μg g^−1^
Keshtkar et al. 2019	*C. indica*	Macro	Ca pretreatment, followed by Xanthation using NaOH and CS_2_	–	X	La, Ce	50–250 mg L^−1^	X	–	2–6	0.5–2.5 g L^−1^	La: 185.44 mg g^−1^ Ce: 172.33 mg g^−1^
Lewis and Guéguen 2022	*E. gracilis*	Micro	–	–	X	Dy	1–100 μg L^−1^	X	–	3–8	0.5 g L^−1^	93 μg g^−1^
Oliveira et al. 2011	*Sargassum sp.*	Macro	Protonation with HCl solution	–	X	Sm, Pr	82–99 mg L^−1^	X	X	5	2 g L^−1^	Sm: 51.1 mg g^−1^ Pr: 40.9 mg g^−1^
Manikandan and Lens (2022)	*Ulva* sp.	Macro	Prior extraction of ulvan in biomass	–	X	La, Nd, Dy	10–1000 mg L^−1^	X	–	7	3 g L^−1^	La: 119.3 mg g^−1^ Nd: 125.3 mg g^−1^ Dy: 128.5 mg g^−1^
Náhlík et al. 2022	*G. sulphuraria*	Micro	–	X	–	Ce, Nd, La, Y	3.3–6.1 mg L^−1^	–	X	3	1 × 10^6^ cells L^−1^	Ce: 26 μg g^−1^ Nd: 15 μg g^−1^ La: 11 μg g^−1^ Y: 11 μg g^−1^
Palmieri et al. 2000	*Monoraphidium* *sp.*	Micro	–	–	X	Nd	4000 mg L^−1^	X	–	1.5	0.46 g L^−1^	1600 mg g^−1^
Palmieri et al. 2002	*S. fluitans*	Macro	Protonation with HCl and H_2_SO_4_ solution	–	X	La	14–972 mg L^−1^	X	–	4–5	2.5 g L^−1^	73.6 mg g^−1^
Pinto et al. 2020a	*U. lactuca, U. intestinalis, F. spiralis, F. vesiculosus, Gracilaria* sp*., O. Pinnatifida*	Macro	–	X	–	Y, La, Ce, Pr, Nd, Eu, Gd, Dy, Tb	89–163 µg L^−1^	X	X	8.2	3 g L^−1^	–
Pinto et al. 2020b			–	X	–	Y, La, Ce, Pr, Nd, Eu, Gd, Dy, Tb	10, 100, 500 μg L^−1^	X	–	8.5	3 g L^−1^	Y: 1047 μg g^−1^ Ce: 938 μg g^−1^ Pr: 1004 μg g^−1^ Nd: 899 μg g^−1^ Eu: 855 μg g^−1^ Gd: 1101 μg g^−1^ Tb: 1193 μg g^−1^ Dy 597 μg g^−1^
Pinto et al. 2022	*Ulva* sp*.*	Macro	–	X	–	Y	20–120 mg L^−1^	–	X	6	3–9 g L^−1^	21.5 mg g^−1^
Ramasamy et al. 2019	*P. oceanica*	Macro	Grafting with a ligand (PAN) using solvent evaporation	–	X	Sc, other REE, heavy metals	1–200 mg L^−1^	X	X	1–5	1 g L^−1^	66.81 mg g^−1^
Sadovsky et al. 2016	*Arthrospira sp.*	Micro	–	–	X	Ce	100 mg L^−1^	X	–	2–5	2.5 g L^−1^	9 mg g^−1^
Sakamoto et al. 2008	*S.hemiphyllum, U. pinnatifida*	Macro	–	X	X	La	6 µg L^−1^	–	X	8	4 g L^−1^ (dw)	0.65 µg g^−1^
Sun et al. 2022	*G. sulphuraria*	Micro	Immobilization by calcium alginate	X	–	La, Y, Sm	40–160 mg L^−1^	–	X	2.5	30% wt	La: 97.2% Y: 96.2% Sm: 98.9%
Viana et al. 2021	*U. lactuca, Gracilaria* sp*.*	Macro	–	X	–	Nd, Dy	10–500 μg L^−1^	X	–	8.5	0.5–5.5 g L^−1^	Nd: 5068 µg g^−1^ Dy: 3366 µg g^−1^
Viana et al. 2023	*Ulva* sp.	Macro	–	X	–	Y, La, Ce, Eu, Gd, Tb, heavy metals	100 μg L^−1^	–	X	7.8–8.0	3 g L^−1^	Y: 382 µg g^−1^ La: 198 µg g^−1^ Ce: 192 µg g^−1^ Eu: 185 µg g^−1^ Gd: 177 µg g^−1^ Tb: 166 µg g^−1^
Vijayaraghavan et al. 2010	*T. Conoides*	Macro	–	–	X	La, Ce, Eu, Yb	99–1005 mg L^−1^	X	–	2–5	2 g L^−1^	La: 154.7 mg g^−1^ Ce: 152.8 mg g^−1^ Eu: 138.2 mg g^−1^ Yb:121.2 mg g^−1^
Vijayaraghavan et al. 2011	*T. Conoides*	Macro	–	–	X	La, Ce, Eu, Yb	97–121 mg L^−1^	–	X	2–5	2 g L^−1^	La: 29.2 mg g^−1^ Ce: 32.2 mg g^−1^ Eu: 31.9 mg g^−1^ Yb: 27.7 mg g^−1^
Vijayaraghavan et al. 2017	*T. Conoides*	Macro	–	–	X	Sm	50–500 mg L^−1^	X	–	2–5	2 g L^−1^	151.62 mg g^−1^

For the size of the sorbent, “Macro” and “Micro” correspond to the use of macroalgae and microalgae, respectively. For the aqueous medium, “Mono” and “Multi” correspond to the use of monocontaminated and multicontaminated systems. Studies which use a given condition of state of the biomass (living or dried) and system contamination (mono or multi), are identified with “X”

#### Role of chemical composition in the sorption process

Depending on their size and chemical composition, algae can be divided into macro and microalgae, each with its characteristics and specificities. Macroalgae are macroscopic, multicellular organisms, which are generally categorized into green (Chlorophyta), red (Rhodophyta) and brown (Phaeophyta) algae, depending on their photosynthetic pigments and cell wall composition (Table 3). Different compounds present in the cell walls of each species group affect the different mechanisms available for REE sorption.

**Table 3:** Biochemical composition, including main polysaccharides of the three macroalgae groups. Adapted from Kostas et al. 2021, Jung et al. 2013 and Ganesh Saratale et al. 2018. Percentages are expressed in dry weight (DW)

Composition	Green algae	Red algae	Brown algae	Microalgae
Ash (%)	18–30	2.5–26	8.7–41	10
Proteins (%)	11–26	10–23	1.1–27	20–60
Lipids (%)	1.0–3.5	0.70–7.4	0.60–3.4	10–65
Carbohydrates (%)	53–70	53–76	34–76	11–47
Main Polysaccharides	Cellulose Hemicellulose Ulvan Starch Mannan	Cellulose Carrageenan Agar Lignin	Cellulose Hemicellulose Laminarin Alginate Fucoidan	
Photosynthetic pigments	Chlorophyll a Chlorophyll b Carotenoids	Chlorophyll a Phycobilin	Chlorophyll a Chlorophyll c Carotenoids	

All groups of macroalgae typically contain high amounts of carbohydrates and low amounts of lipids. While green macroalgae can possess multiple polysaccharides including cellulose and hemicellulose, their distinctive feature is the presence in their cell walls of the soluble ulvan composed of glucuronic acid, xylose, and sulfated rhamnose (Lobban and Wynne 1981). In red algae, the main polysaccharides present in their cell walls are the sulphated polysaccharides carrageenan and agar. Brown algae polysaccharides have a wide range of applications in various industries. Fucoidan (or fucan) is found on the intracellular spaces of brown macroalgal cell walls and is composed of fucose, along with some other sugars such as galactose, xylose and mannose (Sasaki and Yoshikuni 2022). Microalgae have a higher lipid and protein content than macroalgae, which may make these species more interesting in the production of biofuels, rather than applying them as sorbents. It has already been shown that different algae species perform differently when it comes to metal uptake (Lin et al. 2020) due to the different compositions of their cell wall. This is a consequence of the different polysaccharides within each algae species, which provide different functional groups for sorption processes.

In REE sorption with algal sorbents, the analysis of the functional groups involved in the sorption process is usually done using Fourier Transform Infrared Spectroscopy (FTIR). Through FTIR, authors such as Viana et al. (2023) and Manikandan and Lens (2022) state that the hydroxyl (—OH) and carboxylic (—COOH) functional groups present in the ulvan of green algae work as ion-exchangers, favouring REE sorption. Other groups such as —CH, N—H, —C = C and sulfonated bonds from sugars and proteins further potentiate REE sorption. These mechanisms are expected to apply to microalgae as well. Authors such as Lewis and Guéguen (2022) identified several groups involved in the sorption of Nd onto the microalga *Euglena gracilis*. In that study the bands for various polysaccharides, phosphoryl, and amine groups showed shifts after Nd exposure, with the complete disappearance of the C—O—C bond, confirming the binding of Nd to oxygen-rich groups. This shows that the abundance of functional groups available in algae for the binding of REE is a major factor influencing the efficiency of the sorption process.

#### Influence of sorbent size

In a metal recovery application, the first major factor influencing the choice of species should be the size of the sorbent – macro or micro – with which the process will be conducted. Birungi and Chirwa (2014) for 140 mg L^−1^ of La, Palmieri et al. (2000) for 400 mg L^−1^ of Dy and Sadovsky et al. (2016) for 100 mg L^−1^ of Ce, showed that maximum sorption capacities of microalgae species such as *Desmodeus multivariabilis*, *Monoraphidium* sp. and *Arthrospira* sp. can reach hundreds of mg g^−1^ from very concentrated solutions, which generally exceed those tested by studies with macroalgal sorbents. This may be a consequence of the substantially higher surface area and lower amount of microalgae used, compared to macroalgal sorbents. These large uptake capacities are counterbalanced by complications when obtaining the microalgae in the harvesting stage. Small size and low density coupled with electrostatic stability in solution due to their negatively charged surface require additional separation processes that represent additional costs not required in macroalgal cultures (Ge and Champagne 2017). Large-scale processing is further difficulted by the low dry mass content produced in microalgae cultures (< 1% for the commonly used *spirulina* and *chlorella*) (Sebök and Hanelt 2020).

#### Influence of the state of biomass

The use of living macroalgae is less common than dried macroalgae sorbents, justified by the sensitivity of living algae to solution parameters such as pH and metal concentration. Most studies conducted with living macroalgae are experimentally coherent (relative to the sorbent mass, element concentration and solution parameters), and assessed uptake efficiency in pH equal to or similar to that of seawater (7.8—8.5). This can be detrimental to the stability of REE in solution as these elements tend to hydrolyse at high pH and form insoluble hydroxides (Gupta et al. 2019). Furthermore, since most industrial leachates are typically strongly acidic, their application in a restricted pH range will certainly hinder a broad application of living algae in REE recovery. However, when applied to suitable contaminated wastewater or effluent (a non-lethal environment), macroalgae are expected to grow rapidly and create new binding sites, resulting in lower residual concentrations. Concerns about REE toxicity influenced the lower initial concentrations chosen in studies performed with living macroalgae. Despite this, Jacinto et al. (2018) registered no changes in relative growth rates between control and contaminated solutions, suggesting that a multi-contaminated solution containing 0.5 mg L^−1^ of Y, Ce, Nd, Eu and La (2.5 mg L^−1^ of total REE) did not induce significant toxic effects on the species *Gracilaria gracilis*. Costa et al. (2020a) also reported no visual changes in a group of six macroalgae species (*Ulva lactuca*, *Ulva intestinalis*, *Fucus spiralis*, *Fucus vesiculosus*, *Osmundea pinnatifida* and *Gracilaria* sp.) in the presence of nine REE and six potentially toxic elements in a broad ionic strength range. Henriques et al. (2021) also reported that the defence mechanisms of living algae were able to avoid cellular damage when exposed to 500 µg L^−1^ of Eu, along with no growth inhibition. These findings indicate that macroalgae are relatively resistant to adverse effects induced by REE and potentially toxic elements, which are a combination of elements often present in industrial leachates from the E-waste recycling industry. This is an advantageous property, as some of these REE have already been shown to induce oxidative stress on marine fauna when exposed to high concentrations of La and Gd (Henriques et al. 2019; Pinto et al. 2019). Differences in the maximum sorption capacity between living and dried biomass are of great interest from an application point of view. So far, only Sakamoto et al. (2008) presented a comparative study evaluating the La sorption capacity living and dried *Undaria pinnatifida*. With a sorbent dosage of 0.8 g L^−1^ and an initial concentration of 6 µg L^−1^, the differences in the maximum sorption capacity between fresh and dry sorbents were minimal. However, differences in sorption kinetics were noted, as dried biomass reached equilibrium faster than living biomass.

Living algae represent a simpler application of the sorbent since inactivated biomass requires several steps for its preparation. From the studies currently available, it appears that oven-drying, crushing, and sieving are required steps to prepare non-living macroalgae biomass sorbents. However, the use of dried biomass allows for further pre-treatment that has been shown to improve REE sorption efficiency. Simple protonation with acid washing was used by Oliveira et al. (2011) and Palmieri et al. (2002), but also other treatments such as Ca-loading and even grafting with separate ligands have been reported by Diniz and Volesky (2005) and Keshtkar et al. (2019). Of these studies, only Keshtkar et al. showed a comparison of the results obtained for raw and modified algae (*Cystoseira indica*). An increase in sorption efficiency of about 10% was observed for La and Ce after modification (initial concentration of 100 mg L^−1^), showing that the pre-treatment of the biomass promoted REE sorption. In another case, Sun et al. (2022) provided a detailed description of the sorption process when immobilizing the extremophile microalga *Galdieria sulphuraria* in calcium alginate to remove three REE (Y, La, Sm). The authors noted a significant release of Ca^2+^ during the sorption process, leading them to hypothesise that sorption occurs in two distinct phases: initial adsorption based on the affinity of REE towards the available functional groups, and a second phase based on ion exchange in which REE are exchanged with the Ca^2+^ from the calcium alginate. This shows how functionalization and pre-treatment can complement the already high affinity of algal sorbents towards REE, however, it will also become an additional process with associated costs, which may affect the feasibility of a realistic application.

#### Effect of sorbent and solution parameters in removal efficiency and sorption kinetics

The feasibility of algal sorbents in the recovery of REE is not entirely dependent on the sorption efficiency (percentage of removal or final concentration in biomass). In a realistic application, sorption kinetics is also an important aspect of recovery technology. Even if exceptional removal efficiency is achieved, the process may become unattractive if an exceedingly large amount of time is required to achieve it. From the studies available in the literature, it appears that the kinetics of REE sorption onto algal sorbents are influenced by sorbent-related parameters and solution-related parameters. The most intuitive parameter affecting sorption speed is the amount of sorbent used. In theory, an abundance of sorbent implies an abundance of available binding sites for the binding of REE. Consequently, it may be expected that sorption kinetics is also affected by this abundance of binding sites. However, Viana et al. (2021) reported no significant changes to the sorption kinetics of Nd and Dy on the surface of fresh *U. lactuca*, despite an increase in sorbent dosage from 0.5 to 5.5 g L^−1^. Still, the amount of sorbent to be used will depend on the circumstances. If maximum removal is desired, more sorbent should be used, but if concentrating the REE in the sorbent is the objective, lower amounts may be more appropriate. For example, Pinto et al. (2022) evaluated the influence of sorbent dosage (3 – 9 g L^−1^) in the removal of Y by *Ulva* sp., achieving the best removal for the highest mass of sorbent. However, the spreading out of the REE in the abundant binding sites resulted in a less enriched sorbent. Similarly, Keshtkar et al. (2019) evaluated the effects of different sorbent dosages and concluded that a minimal amount (0.5 g L^−1^) of the sorbent is preferred to saturate the maximum amount of available binding sites. Regarding algal sorbents, the major sorbent-intrinsic factor influencing the sorption kinetics is the state of the biomass (i.e. living or dried sorbents). By analysing the available studies in the literature, it appears that dried sorbents reach an equilibrium in a significantly lower amount of time. Among the kinetic studies available with dried sorbents, the results are surprisingly coherent. Vijayaraghavan et al. (2010), Sadovsky et al. (2016), Keshtkar et al. (2019), Oliveira et al. (2011) and Manikandan and Lens (2022), all reported an equilibrium time between 100 and 150 min. Birungi and Chirwa (2014) and Palmieri et al. (2002) reported equilibrium times between 50 and 100 min. Kinetic curves are usually characterized by a sharp increase in the first minutes of contact, followed by an attenuation of the curve until 100 – 150 min. All of these studies used similar sorbent dosages (2 – 3 g L^−1^), except for Birungi and Chirwa (2014) (0.5 g L^−1^). The fact that the sorption kinetics of dried sorbents are so coherent regardless of the species used, the element to be sorbed, and the initial element concentration facilitate the reproducibility of the technology in a wide array of experimental conditions. As for living sorbents, these showed much slower sorption kinetics than dried sorbents, often exceeding several days to reach a pseudo-equilibrium. Jacinto et al. (2018), Pinto et al. (2020b), Ferreira et al. (2020), and Henriques et al. (2021) all collectively used living macroalgae to remove 500 µg L^−1^ of different REE from contaminated solutions. All of these studies applied Pseudo-First Order (PFO) and Pseudo-Second Order (PSO) curve fittings to their results and observed that equilibrium was not reached before 48 h, regardless of algae species and element to be sorbed. Kinetic curves are usually characterized by a sharp increase in the first few hours of contact, followed by an attenuation of the curve until 48 – 72 h. It may be possible that a lack of equilibrium is caused by processes such as the living sorbent’s growth and bioaccumulation processes, which create new binding sites. However, the time required for these parameters to take a significant part in the sorption process may not be attractive in practical terms.

As per the solution parameters, the initial REE concentration and its influence on kinetics are crucial in the sorption process. Manikandan and Lens (2022) studied the sorption of La, Nd and Dy by *Ulva* sp. biomass and discovered that, while the sorbent became more enriched for higher concentrations, the sorption kinetics did not suffer significant alterations for initial concentrations ranging from 10 to 1000 mg L^−1^. Similar results were obtained by Vijayaraghavan et al. (2017), using the dried macroalga *T. conoides* to remove different concentrations of Sm (50, 100 and 200 mg L^−1^) from contaminated solutions. This shows that the kinetics curves of REE sorption onto algal sorbents are independent of the initial concentration gradient, which means that in future applications, the initial concentration may be increased without negatively impacting the process. As per the influence of pH on the sorption process, it was already stated that living biomass possesses a limited pH window (close to that of seawater) to be an effective sorbent. However, studies using dried biomass have explored wider pH windows, often between 2 and 6. Diniz and Volesky (2005), Iovinella et al. (2022), Keshtkar et al. (2019), Palmieri et al. (2002), and Vijayaraghavan et al. (2011) all applied dried algal sorbents to remove REE from spiked solutions at different pH values (2 – 6) and reported a common behaviour: the sorption efficiency reaches a peak at pH 5, progressively decreasing with the decrease of pH, reaching minimum efficiency at pH 2. This reveals not only the similar functional groups among algae species but also a clear influence of pH on the surface properties of the biosorbents, such as their surface charge. The surface charge of an adsorbent can be measured through electrochemical methods. While the previously mentioned studies did not perform any electrochemical analysis, Lewis and Guéguen (2022) measured the zeta potential of *E. gracilis* cells in Milli-Q water, identifying its isoelectric point at a pH of 2.3. Assuming that the functional groups in *E. gracilis* are similar to those in the previously mentioned studies, this may help understand the pattern of the pH-dependent behaviour. The closer the pH is to 2.3, the less electronegative the surface becomes, thus “repelling” the positive REE^3+^ ions.

The sorption behaviour of a sorbent can be strongly affected by other competing ions present in the solution. An efficient sorbent in a mono-contaminated system can perform poorly in a multi-contaminated system due to poor selectivity and competition among other ions present in the solution. This is especially true for REE as they share similar chemical properties and therefore similar binding mechanisms. Studies by Ramasamy et al. (2019) and Pinto et al. (2020a) describe a preference for LREE in the absence of competing ions, which was lost in multi-contaminated systems. This competition effect tends to be less pronounced in HREE, possibly due to reduced ionic radius and poor shielding of f-subshells (lanthanide contraction). The study conducted by Jacinto et al. (2018) showed an increase in sorption for La, Ce, Nd and Eu (all of them LREE) and a decrease in Y (considered an HREE) when all of these elements were present in the same solution. Vijayaraghavan et al. (2011) also showed an antagonistic effect among REE in a multi-contaminated system. This variability in competition effects underscores the need to study this phenomenon on a case-by-case basis, as some different algae may have different affinities for REE.

#### Algae and rare earth elements recovery from real wastes

Their wide availability and abundance make algae a cheap sorbent with general characteristics of high surface area and sorption capacity (SarI and Tuzen 2008; Jayakumar et al. 2015). However, as stated previously by Tofan et al. (2022) and Gupta et al. (2019), realistic applications of these sorbents are still sorely lacking in the literature. For all methodologies, the transition from a laboratory to an industrial scale is an inevitably challenging process. This is a particularly challenging task when the research field is still in its infancy, as is the case of REE preconcentration in algal sorbents. So far, most studies have only focused on idealistic scenarios using synthetic solutions with easily manipulable parameters such as pH, metal concentration and ionic strength. A few exceptions include a study by Pinto et al. (2020a, b) who used *Ulva* sp. to remove Y from a diluted waste (fluorescent lamp waste) and reported a decrease in sorption efficiency (%) compared to previous studies. Similarly, Ramasamy et al. (2019) reported a significant reduction in sorption efficiency using an algal sorbent in a semi-realistic waste (REE-spiked acid-mine drainage solution). As such, more studies are required to assess the potential of algal sorbents to be used in realistic scenarios. The steady advances in algae sorbent research have only shown that they are a promising alternative to other expensive and polluting methods of enriching these elements. Major challenges are to be expected such as unfavourable pH values due to acidic extraction processes, the presence of high concentrations of potentially toxic elements, large variability and unpredictability of matrix conditions and the toxicity of the “treated” effluent obtained after REE sorption.

## Key parameters for large-scale application of algal-based sorbents

### Potential for carbon capture

Algal sorbents may offer other advantages compared to other sorbents, the most important being carbon fixation during photosynthesis. As climate change is a growing concern, measures have already been established to limit the rise in global temperature to 2 °C above pre-industrial levels. These measures are mostly based on the reduction of CO_2_ emissions (Moreira and Pires 2016). Algae provide the unique characteristic of photosynthesis, converting sunlight and atmospheric CO_2_ into organic carbon and increasing biomass (Farrelly et al. 2013). It is known that this CO_2_ bio-fixation contributes to a reduction in the excess CO_2_ available in the air and a minimization of the resulting climate changes (Chew et al. 2018). If algae-based sorbents are to be used in a REE recovery process, the potential role of photosynthesis in a carbon–neutral process should not be ignored. This characteristic may dictate the implementation of such a process. Studies which quantify assimilated carbon during algae production show that 1 kg of produced algae biomass consumes approximately 1.88 kg of CO_2_ (Adamczyk et al. 2016). While algae can capture CO_2_ from natural sources, combustion exhaust also represents a viable secondary source that can be directly injected into large-scale cultures. Under these circumstances, algae would act as an afterburning tool to sequester CO_2_ that would otherwise be released into the atmosphere, thus contributing to reducing greenhouse gas emissions (Molazadeh et al. 2019).

### Cultivation methods

**Fig. 1:**
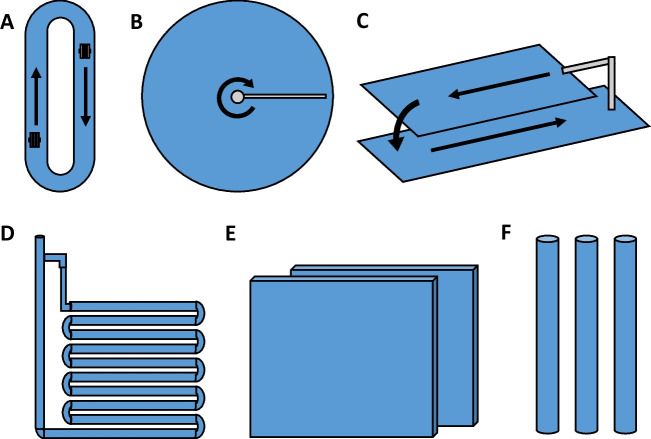
Schematic representation of the different inland algae cultivation systems. A-C refer to open-air systems, while D-F are closed systems. Individually, they are referred to as: A—Raceway system, B—Circular system, C—Cascade System, D—Tubular system, E—Flat system, F—Column system. Adapted from Voloshin et al (2016)

To increase the feasibility of REE recovery using algal-based sorbents, cost-effective and efficient large-scale production of algal feedstocks is required. The typical inland algal cultivation systems can be divided into open-air systems and closed systems, so-called photobioreactors (Fig. 1).

Each of these systems presents various advantages and disadvantages (Table 4) and a careful assessment is required, considering the algae species, climactic conditions and the cost of maintenance.

**Table 4:** Advantages and disadvantages of different algae cultivation systems

System		Advantages	Disadvantages
Open-air	Raceway	• Low construction cost • Low operational cost • Easy maintenance • Simple cultivation • High scale-up capacity	• Easily contaminated • Sensitive to weather effects • Large amounts of wastewater • Photoinhibition
	Circular	• Low price • Easy maintenance • Efficient mixing • High scale-up capacity	• High energy expenses • Limited size • Easily contaminated • Sensitive to weather effects • Large amounts of wastewater
	Cascade	• Efficient mixing • High photosynthetic potential • Easy maintenance • Low price	• Easily contaminated • Sensitive to weather effects
Photo-bioreactors	Tubular	• Low investment • High surface area • High yield • Easily controlled conditions	• Photoinhibition • Hard to clean • Gradients of O_2,_ CO_2_ and pH along the tubes • Expensive harvesting
	Flat	• Exceptional surface area • Low maintenance cost • Easy to maintain • Short oxygen path	• Difficulties in scale-up • Stress/damage to some algae strains • Difficulties in temperature control • Expensive harvesting
	Column	• Low energy consumption • Efficient mixing • Reduced photoinhibition • Easy sterilization • Low shear stress	• Small surface area • Expensive • Shear stress to some algae strains • Difficulties in scale-up • Expensive harvesting
Off-shore		• Reduce the impact of surrounding aquacultures • Efficient harvesting techniques • High scale-up capacity	• Limited number of species • Potential disruption of marine ecosystems • Risk of nutrients depletion and eutrophication

Open-air systems may include natural ponds, but these are only established in locations where climate, salinity and pH conditions are optimal. Therefore, the most common open-air systems are those that can be installed manually with specific features that maximize growth. These include mainly raceway systems, circular systems, and cascade systems. Raceway systems consist of multiple loopback ponds in which paddle wheels cause water to flow. Circular systems are cylindrical shallow ponds in which stirring is induced by a central rotor connected to a pivoting agitator. While raceway and circular systems are suitable for the cultivation of macro- and microalgae, cascade systems focus on the cultivation of microalgae and utilize thin inclined water in which turbulence is created by the action of gravity (Voloshin et al. 2016). Open-air systems still have some disadvantages such as sensitivity to adverse weather events, limited light availability and lack of temperature control. In addition, contamination by other heterotrophic organisms is almost inevitable, resulting in a heterogeneous harvested biomass (Nigam and Singh 2011). Closed photobioreactors enable constantly optimal conditions for algae growth while avoiding contamination at the same time, but at the expense of higher energy consumption. In these cases, cellular damage through mechanical agitation is also avoided. In general, photobioreactors are used for microalgae cultivation and can be divided into three types: tubular, flat and column photobioreactors. Tubular systems consist of a series of typically serpentine glass tubes in which the water flows with the help of a pump. A heating system helps maintain an even temperature along the pipes and CO_2_ is provided using an injection system. This system is relatively cheap and suitable for outdoor cultivation. However, temperature and pH gradients along the tubes and some wall growth can cause undesirable consequences such as photoinhibition (Molina et al. 2001). Flat photobioreactors, in which medium flows between the transparent glass surface, have the highest photosynthetic potential due to their large surface area. They are relatively cheap and use less energy than tubular photobioreactors. However, wall growth and difficulties with scale-up and temperature control can pose an obstacle to large-scale cultivation (Voloshin et al. 2016). Finally, column photobioreactors are cylindrical tubes in which the medium is aerated from the bottom. Although expensive, they provide efficient mixing, lower energy consumption, reduced photoinhibition, low shear stress and are also easier sterilization. Still, diminished illumination surface area may occur in large scale-ups.

Cultivation of microalgae typically yields a dilute solution (0.02 – 0.05% solids), and efficient harvesting methods are one of the major challenges in large-scale microalgae cultures (Zamalloa et al. 2011). Separating the microalgae from the suspension into a slurry or paste with concentrations between 5 and 25% of total solids (bulk harvest), followed by a second concentration method that can increase the biomass content up to 99% (thickening) (Abdelaziz et al. 2013). Due to the properties of the microalgae in the solution, this process step can account for up to 30% of the total production costs. Microalgae concentrates are composed of organisms of different sizes, shapes and mobility that can influence harvesting processes (Uduman et al. 2010). The major harvesting techniques for microalgae include sedimentation, flotation, filtration, centrifugation and flocculation (Abdelaziz et al. 2013). Sedimentation uses gravity to separate two components with different densities. Although useful in wastewater treatment, this is a very time-consuming process in algae harvesting since microalgae are typically very similar in density to water. Similarly, flotation is a gravity separation process, but in the opposite sense, where gas (or air) is introduced into the mixture, adhering to the particles, and bringing them to the surface. Auto-flotation is a viable option for microalgae cultures since oxygen produced by photosynthesis facilitates harvesting (Koopman and Lincoln 1983). Filtration is more commonly used at the laboratory scale because membrane clogging, and high maintenance costs can hamper its application on a larger scale. On the other hand, large-scale centrifugation is a very fast and efficient process, but it is significantly more energy intensive. Flocculation is based on the aggregation of suspended particles by adding a flocculant. Since the negatively charged algal surface naturally prevents the aggregation of microalgae cells, this technique efficiently counteracts this property. Nevertheless, flocculants are usually used in large quantities without guaranteed sustainable production. In addition, flocculants are pH sensitive, selective for some species, and can induce contamination of harvested biomass. Recently, research on biodegradable and environmentally friendly flocculants such as chitosan and cationic guar gum have offset some of these concerns (Riaño et al. 2012; Banerjee et al. 2013).

With mass cultivation inland, large areas that are otherwise non-arable can be used. However, large water and nutrient supplies (mainly nitrogen and phosphorous) are required, which can account for up to 60% of the total biomass production costs. To counteract these operational costs, some authors suggested including nutrient-rich wastewater (such as urban and industrial streams) in the algal cultivation stage. During growth, algae assimilate the nutrients available in the wastewater, adding value to it and contributing to a zero-waste concept (Choudhary et al. 2020). Still, the desired characteristics of the algal biomass can be compromised when wastewater streams are used since algae are highly responsive to culture medium conditions. This is due to the stoichiometric flexibility of algal cells, which allows the nitrogen/phosphorous ratio to be proportional to that in the culture medium. Therefore, the use of certain wastewaters in algal culture should be evaluated on a case-by-case basis. Regarding advances in algal cultivation using wastewater streams, most studies focus on the microalga genus *Chlorella*, but other common genera include *Scenedesmus*, *Phormidium*, *Chlamydomonas*, *Nannochlorpsis* and *Nitzchia* in municipal and agro-industrial wastewaters (Abdelaziz et al. 2013; Choudhary et al. 2020). But even if the costs associated with water availability were reduced, energy requirements, harvesting and processing would still represent a major cost in the process. In turn, algal cultivation in off-shore (or near-shore) systems can be an important source of algal biomass without the limitations of water and energy supplies. They are intended solely for the cultivation of macroalgae species as it is not feasible to contain microalgae in an offshore environment. The most commonly grown macroalgae belong to the red and brown categories, with green macroalgae accounting for only 20% of the total harvest. Current farming techniques are based on the artificial cultivation of seedlings in a substrate such as a rope, under controlled greenhouse conditions, after which they are transplanted to the farming site where they grow into fully harvestable algae. The most promising macroalgae species are *Laminaria japonica*, *Undaria pinnatifida*, *Euchema spp.*, *Kappaphycus alvarezzi* and *Gracilaria Verrucosa* (Jung et al. 2013). Of these species, those belonging to the *Laminaria* genus, commonly known as kelp, account for nearly a third of total macroalgae production.

Some studies argue that offshore macroalgae cultivation could also be integrated with other aquaculture to add value and reduce environmental impact. Fish aquacultures are known to produce high concentrations of nitrogen and phosphor (derived from the excretion process) that can cause eutrophication phenomena. By coupling nearby algal cultures, these nutrients could be taken up by the algae, reducing the environmental impact and the cost of biomass production (Filote et al. 2021). Harvesting macroalgae in offshore farms is a far more efficient process than those available for microalgae cultures. Manual harvesting is widespread and effective, however, the concept of large-scale macroalgae farming has always been associated with species-specific mechanical harvestings techniques like mowing, suction or dredging. For example, in the case of *L. japonica*, growth structures may need to be transported to the shore, while in the case of the upright-growing *Macrocystis pyrifera*, mowers deployed from ships may be used (Roesijadi et al. 2010).

### Integral use of the biomass post-sorption

**Fig. 2:**
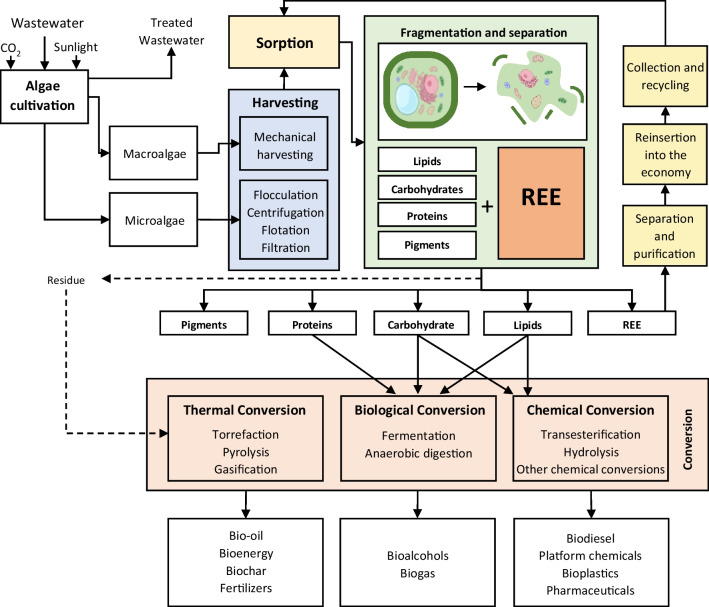
Integrated use of algae biomass incorporated into a REE recovery process

The biomass yield obtained from bulk algal cultures would be used for sorbent production, however, after sorption, other uses for the spent biomass could be considered as algae are also regarded as a potential sustainable source of green energy, biofuel and several platform chemicals (Chew et al. 2021). Integrated use of the algal biomass after sorption can contribute to the economic feasibility of the sorption process. Only the full utilization of biomass together with the integration of upstream and downstream processes is economically feasible (Kannah et al. 2021). A hypothetical representation of an integrated REE recovery process using algae is proposed in Fig. 2. In this example, wastewater is included in algae cultivation, as is CO_2_ captured from sources such as flue gas. Wastewater serves as a source of nutrients for the algae, while the algae serve as a treatment process after which the treated wastewater can be reused.

After sorption, the separation of the algal components represents the most challenging step of the process, along with the isolation of the REE. Although this approach has not yet been attempted, authors have previously suggested the possibility of separating REE from the different algal cell organelles by cell fractionation (Cao et al. 2021). After fractionation, the various components can undergo biological and chemical transformation to produce high-value products. Any residue resulting from these conversion processes can be recovered and subjected to thermochemical conversion to achieve full biomass utilization. All process steps must optimize parameters such as algae growth conditions, energy requirements, water consumption and infrastructure.

### Prospects for the industrial application of algal-based sorption to recover REE

Adding the potential of REE recovery to the already established usefulness of algae in sectors such as the global food and chemical markets would represent another contribution to a sustainable and blue economy. However, the economic feasibility of marine biorefineries themselves is heavily affected by the cost of biomass production. According to previous reports, producing 1 ton of macroalgae biomass (Dry Weight, DW) may cost between 500 $ and 700 $ (Valderrama et al. 2013), most of which is currently used for food purposes. Extracted products sell for one order of magnitude more. As such, additional costs of parallel cultivation exclusively for biosorbent purposes may prove too expensive to be successfully incorporated into a marine biorefinery concept. These costs may be counteracted by using leftover biomass from food-purposed cultivation. Only 8—12% of cultivated algae is used for food and bioproducts, while the remaining biomass is disposed of as waste (Lehahn et al. 2016). Since sorbent biomass does not need to fulfil the quality requirements expected for algae-based foods, this may represent a window of opportunity to add value to cultivation wastes which are often incinerated or used as fertilizers. The idea that algae wastes can be used as biosorbents has already been mentioned in previous reviews such as Filote et al. (2021). This very concept has already been applied by Filote et al. (2019), who used biorefinery waste of *Fucus spiralis* to remove the toxic metal Pb from contaminated waters. The concept of algae-based sorbents derived from biorefinery wastes for the recovery of REE would later be explored by Manikandan and Lens (2022). The authors extracted ulvan from *Ulva* sp. and used the resulting residue as a biosorbent to remove La, Nd and Dy from synthetic solutions. This residue was revealed to be more efficient than the whole biomass, allowing for a REE recovery rate of over 90%. Despite this, no algal biorefinery wastes have yet been applied to recover REE from real wastes. This transition would certainly impose many challenges since additional processes such as the extraction of the REE from the wastes and the transportation costs of biomass to the sorption site would have to be taken into account. As such, a life cycle assessment (LCA) should be carried out in the future to determine the profitability and the influence of the individual processes on the overall technology. Given the early stages of the development of algae-based sorption technologies, it can be assumed that an integrated approach to using algae in a REE recovery process in conjunction with other uses such as biofuel production is still far from a reality. However, if successfully applied, this technology could represent a catalyst for fully integrated and sustainable marine biorefineries, as well as promote the circularity of critical resources.

## Conclusion

The current stage of REE recovery technologies shows the need to look for sustainable and environmentally friendly alternatives. While efficient, pyro- and hydrometallurgical processes display negative impacts that on the environment which need to be addressed. Furthermore, the depletion of natural resources combined with the high criticality of REE requires a technology that can efficiently recover these elements from secondary sources from which E-waste seems most promising. Algae-based sorbents show promise in removing these elements from aqueous media, representing a unique and environmentally friendly approach to the recovery of these elements, as shown by the numerous sorption studies reviewed in this work. These studies have evaluated the efficiency of an array of different algae species, under numerous different experimental conditions. The variability of experimental parameters among studies is not only justified but also required for a deep understanding of the sorption process, due to the nature of algal sorbents and the possibility of their application under various forms. Those which apply living sorbents are coherent with each other in terms of initial REE concentration and sorbent dosage, despite the numerous species tested. Another similarity among these studies is the pH range of 6 – 8, which is a consequence of the limitation of using living organisms as sorbents. Such a limitation may hinder their application in strongly acidic scenarios, which is often the case with waste-leaching solutions. However, easy separation methods post-sorption (in comparison to dried/microalgal sorbents) and high affinity towards most REE may prove useful in applying living algal sorbents in the recovery of REE from contaminated aquatic systems, rather than from a specific waste. Dried sorbents were successfully applied in more acidic conditions and for higher initial REE concentrations, which makes them attractive in a potential process for the recovery of REE from real wastes. While previous studies established the foundations of REE recovery using algal sorbents, the potential for a realistic application at an industrial level is still uncertain. Numerous parameters which affect the efficiency of these sorbents have already been studied, such as pH, and salinity, but only in laboratory-scale experiments (which seldom transfer to a realistic scenario). Most previous works have studied the sorption process in idealized scenarios, making the behaviour of these sorbents in a real application unpredictable. In truth, the competitive effect between specific REE and other cations reported by some of these works may indicate a potential loss in efficiency in the transition to more complex matrices. From today’s perspective, evaluating the efficiency of these sorbents in real (complex) scenarios seems the next logical step in this field of research. Nevertheless, the use of algae-based sorbents has inherent advantages from an environmental point of view. Carbon capture during algae cultivation is a captivating feature in terms of achieving a carbon–neutral process, and the many potential uses of algal biomass such as added-value bioproducts add to the already established notion that these organisms could be the key to sustainable development and a green economy.

## Data Availability

Not applicable
